# Supplementary material for: Proteogenetic drug response profiling elucidates targetable vulnerabilities of myelofibrosis
Source: Nat Commun. 2023 Oct 12;14:6414. doi: 10.1038/s41467-023-42101-z (PMC10570306; doi:10.1038/s41467-023-42101-z)
Supplement: Supplementary file 1 — Supplementary Information [file 41467_2023_42101_MOESM1_ESM.pdf]

Supplementary Information

Proteogenetic drug response profiling elucidates targetable vulnerabilities of myelofibrosis

Mattheus H.E. Wildschut[1,2,3], Julien Mena[1], Cyril Dördelmann[4], Marc van Oostrum[2], Benjamin D. Hale[1], Jens Settelmeier[2,5], Yasmin Festl[1], Veronika Lysenko[3], Patrick M. Schürch[3], Alexander Ring[3], Yannik Severin[1], Michael S. Bader[6], Patrick G.A. Pedrioli[2,5,7], Sandra Goetze[2,5,7], Audrey van Drogen[2,5,7], Stefan Balabanov[3], Radek C. Skoda[6], Massimo Lopes[4], Bernd Wollscheid[2,5,*,✉], Alexandre P.A. Theocharides[3,*,✉], and Berend Snijder[1,*,✉]

[1]Institute of Molecular Systems Biology, Department of Biology, ETH Zurich, Zurich, Switzerland
[2]Institute of Translational Medicine, Department of Health Sciences and Technology, ETH Zurich, Zurich, Switzerland
[3]Department of Medical Oncology and Hematology, Division of Hematology, University Hospital Zurich, Zurich, Switzerland
[4]Institute of Molecular Cancer Research, University of Zurich, Zurich, Switzerland
[5]Swiss Institute of Bioinformatics, Lausanne, Switzerland
[6]Department of Biomedicine, Experimental Hematology, University Hospital Basel and University of Basel, Basel, Switzerland
[7]ETH PHRT Swiss Multi-Omics Center (SMOC), Switzerland
* Co-senior and co-corresponding authors
✉ Correspondence to bernd.wollscheid@hest.ethz.ch, alexandre.theocharides@usz.ch or bsnijder@ethz.ch

Inventory of Supplementary Information

Supplementary Information 1
Inventory of Supplementary Information 1
SUPPLEMENTARY FIGURE LEGENDS 3
Supplementary Figure 1: Development of single-cell oncogenic imaging readouts based on CALRm and pSTAT5 immunofluorescence 3
Supplementary Figure 2: Image-based CNN cell type identification and quantification of MF PBMC samples 5
Supplementary Figure 3: Pharmacoscopy drug responses of the MF PBMC cohort 7
Supplementary Figure 4: Clinical proteotyping quality controls 9
Supplementary Figure 5: Large-scale MPN granulocyte proteotyping 11
Supplementary Figure 6: Characterization of the proliferative MCM-high MF phenotype 13
Supplementary Figure 7: Characterization of homozygous *CALR* mutation-induced ER stress 15
Supplementary Figure 8: Graphical summary of the study workflow and key findings 16
SUPPLEMENTARY TABLES 17
Supplementary Table S1: The full clinical annotations of the different included cohorts. 17
Supplementary Table S2: The processed pharmacoscopy drug response matrices. 17
Supplementary Table S3: The processed proteotype matrices. 17
SUPPLEMENTARY REFERENCES 17

Supplementary Figure 1

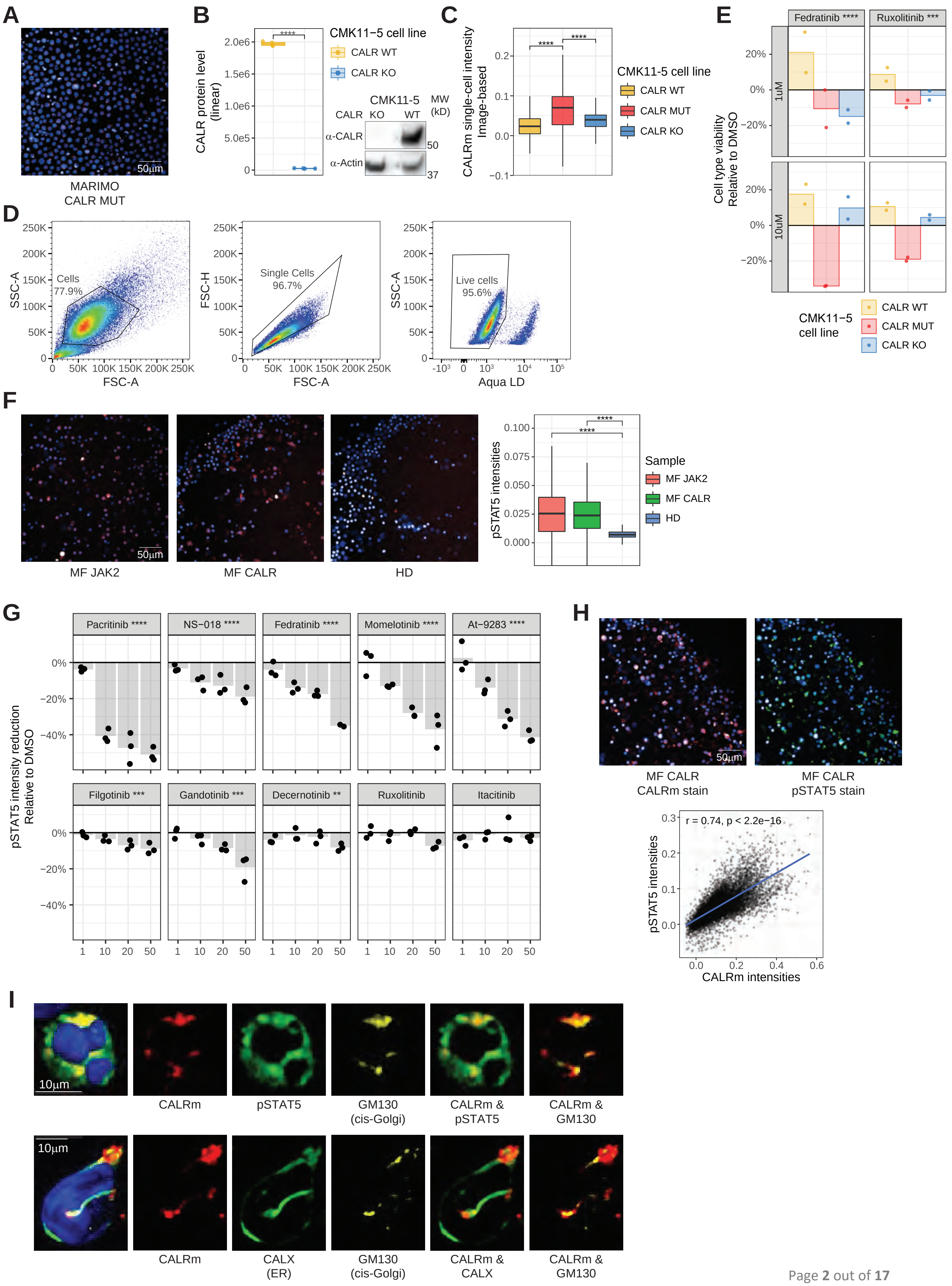

SUPPLEMENTARY FIGURE LEGENDS

Supplementary Figure 1: Development of single-cell oncogenic imaging readouts based on CALRm and pSTAT5 immunofluorescence

(A) Staining of the *CALR*-mutated MARIMO cell line with the CALR mutant-specific antibody CALRm. A representative image is shown (left panels; blue: DAPI; red: CALRm).

(B) Validation of the CRISPRed CMK KO cell line, by DIA-MS/MS quantification of three replicate measurements per cell type (left panel) and Western Blot (WB; right panel). For WB, Actin is included as a loading control.

(C) Image-based quantification of CMK single-cell mean CALRm intensities. Exemplary images are shown in Figure 1A. Boxplots represent quantification of 4 replicate wells with 25 images per well.

(D) Flow cytometry gating strategy of the CMK11-5 cell lines stained with CALRm as quantified in Figure 1A.

(E) JAK inhibitor (JAKi) drug responses of a mixed *CALR* mutant cell line panel. Wild type parental CALR WT cells are mixed with CALR MUT and CALR KO cells (1:1:1 ratio) and exposed to ruxolitinib and fedratinib. Using pharmacoscopy, relative drug sensitivities of the different cell lines are calculated across two concentrations with two replicate wells per concentration. p-values indicate significance of CALR MUT depletion across replicates and concentrations per drug compared to DMSO-treated conditions.

(F) pSTAT5 staining and single-cell quantification of MF patient and HD PBMCs. Representative images of different PBMC samples are shown (blue: DAPI; red: pSTAT5). Boxplots show single-cell mean intensities calculated across all cells.

(G) pSTAT5 drug responses upon treatment of MF PBMCs with a JAKi drug panel. PBMCs isolated from an MF JAK2 patient were exposed to different drug concentrations with three replicate wells across concentration, after which depletion of single-cell pSTAT5 levels was quantified by pharmacoscopy. p-values indicate significance of pSTAT5 depletion across replicates and concentrations per drug compared to DMSO-treated conditions.

(H) Single-cell quantification of an MF PBMC sample co-stained with CALRm and pSTAT5. The first panel shows DAPI (blue) and CALRm (red) and the second panel shows DAPI (blue) and pSTAT5 (green) of the same field of view. Single-cell mean intensities are calculated and correlated (right panel). Pearson correlations and corresponding correlation significance are reported.

(I) High-resolution imaging of subcellular localization of CALRm and pSTAT5 stains. Average intensity representations of Z-stacks covering the cells are shown. Calnexin (CALX) and GM130 are used as markers for the ER and the cis-Golgi, respectively. Selected channels of the same field of view are shown for two differently stained cells. Representative results of two independent repeats are shown.

Asterisks indicate non-adjusted two-sided Student's t-test significance: **** = $p < 0.0001$, *** = $p < 0.001$, ** = $p < 0.01$, * = $p < 0.05$; exact p-values are reported in Source Data. Boxplots as in Figure 1A.

Supplementary Figure 2

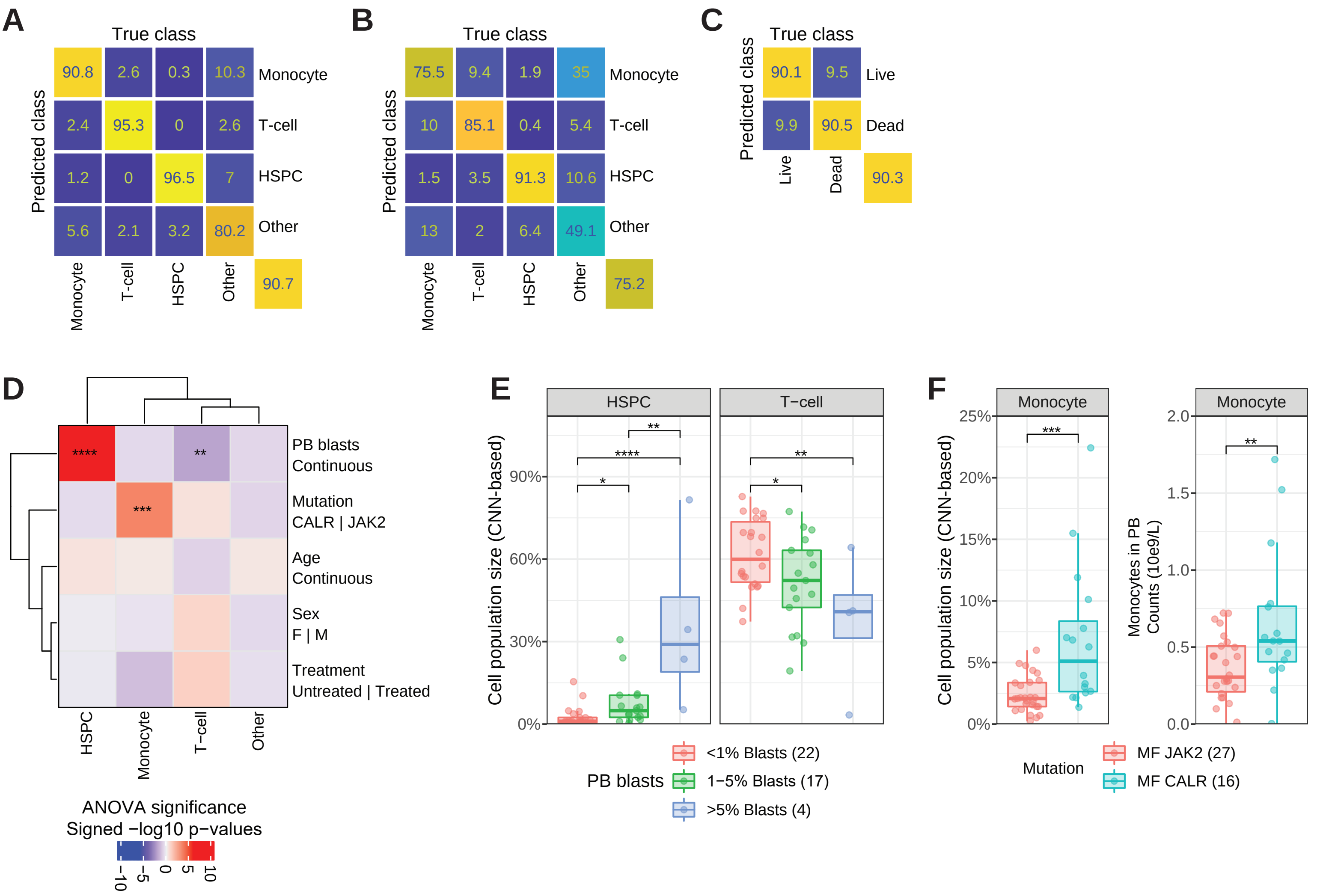

Supplementary Figure 2: Image-based CNN cell type identification and quantification of MF PBMC samples

(A and B) Confusion matrix of the **(A)** image-based and **(B)** feature-based CNN cell type classifiers. Networks were trained on the same single-cell crop input images. Diagonal numbers indicate prediction accuracies per cell type of the disjoint test set not used for training of the CNN. Network accuracies across cell types average to an overall 90.7% and 75.2% accuracy across all included MF PBMC samples for image- and feature-based classification, respectively.

(C) Confusion matrix of the image-based CNN live/dead cell classifier. Diagonal numbers indicate prediction accuracies per cell type of the disjoint test set not used for training of the CNN, averaging to an overall 90.3% accuracy across all included MF PBMC samples.

(D) Associations between cell population sizes and clinical factors as determined by ANOVA across the MF PBMC cohort. For continuous factors (PB blasts and age), red indicates positive associations, and blue negative. For discrete factors (mutation, sex, and treatment) red indicates association to the first term, and blue to the second. p-values indicate ANOVA significance.

(E) MF PBMC cell population sizes of HSPCs and T-cells stratified for PB blast counts. MF patient counts are represented in brackets. p-values indicate Student's t-test significance.

(F) Validation of CNN-based association of monocyte counts with mutation status. CNN-based monocyte cell population fractions (left panel) are compared to clinical PB monocyte counts (right panel) both stratified for MF patient mutation status. MF patient counts are represented in brackets. p-values indicate Student's t-test significance.

Asterisks indicate non-adjusted two-sided significance: **** = $p < 0.0001$, *** = $p < 0.001$, ** = $p < 0.01$, * = $p < 0.05$; exact p-values are reported in Source Data. Boxplots as in Figure 1A.

Supplementary Figure 3

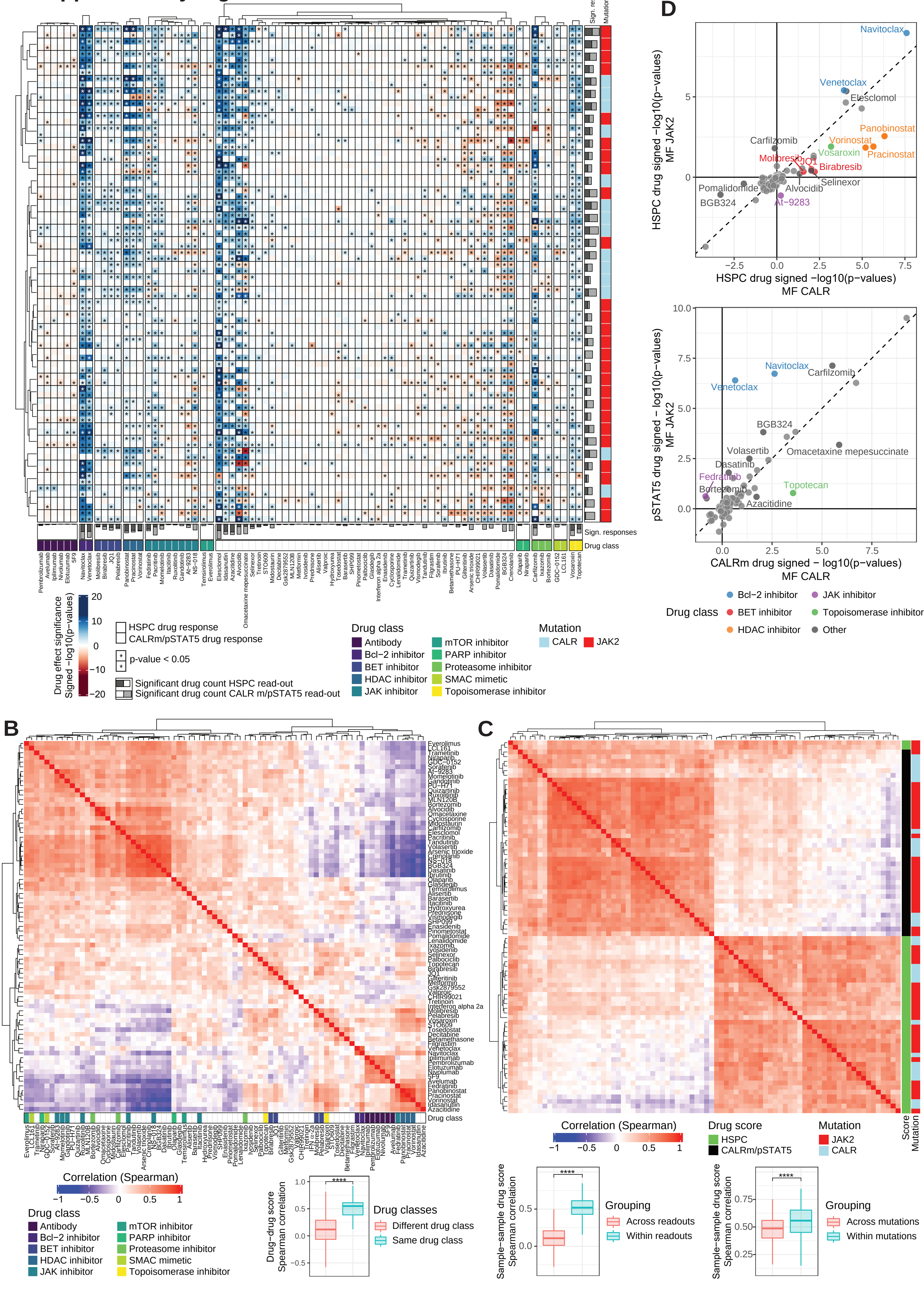

Supplementary Figure 3: Pharmacoscopy drug responses of the MF PBMC cohort

(A) Clustergram of the HSPC and oncogenic CALRm/pSTAT5 drug responses across the MF PBMC cohort. Rows indicate patients and columns drugs. Each individual patient-drug response box shows the HSPC (top) and CALRm/pSTAT5 (bottom) drug effect significance, with colors indicating signed p-values per drug compared to the respective control conditions. Blue indicates on-target depletion and red increase of the respective readout upon drug treatment. Asterisks indicate significant drug responses ($p < 0.05$). Bar graphs summarize the number of significant responses per drug readout per patient (right) or drug (bottom). Right annotations indicate patient mutation status and bottom annotations selected drug classes.

(B) Drug-drug correlation matrix of drug responses across the MF PBMC cohort. Colors indicate Spearman correlations of HSPC and oncogenic drug responses per drug. Bottom annotations indicate selected drug classes. Inset shows boxplot of drug-drug correlations of drugs within the same annotated drug class compared to drug-drug correlations of drugs belonging to different drug classes (n=60 and n=2031 correlations).

(C) Patient-patient correlation matrix of HSPC and oncogenic drug responses across the MF PBMC cohort. Colors indicate Spearman correlations of HSPC and oncogenic drug responses per patient and drug score. Inset box plots show similarity of drug responses within drug scores compared to those across drug scores (left panel; n=3200 and n=3200 correlations) and drug score similarity within the same and across different driver mutations (right panel; n=1456 and n=1744 correlations).

(D) Comparison of mutation-stratified drug responses. Median MF CALR (x-axis) and MF JAK2 (y-axis) HSPC (top panel) and oncogenic (bottom panel) drug responses are shown. Drugs with an absolute difference >1 in the two mutations are annotated and colored per drug class. Dashed line represents the diagonal.

Asterisks indicate non-adjusted two-sided Student's t-test significance: **** = $p < 0.0001$; exact p-values are reported in Source Data. Boxplots as in Figure 1A.

Supplementary Figure 4

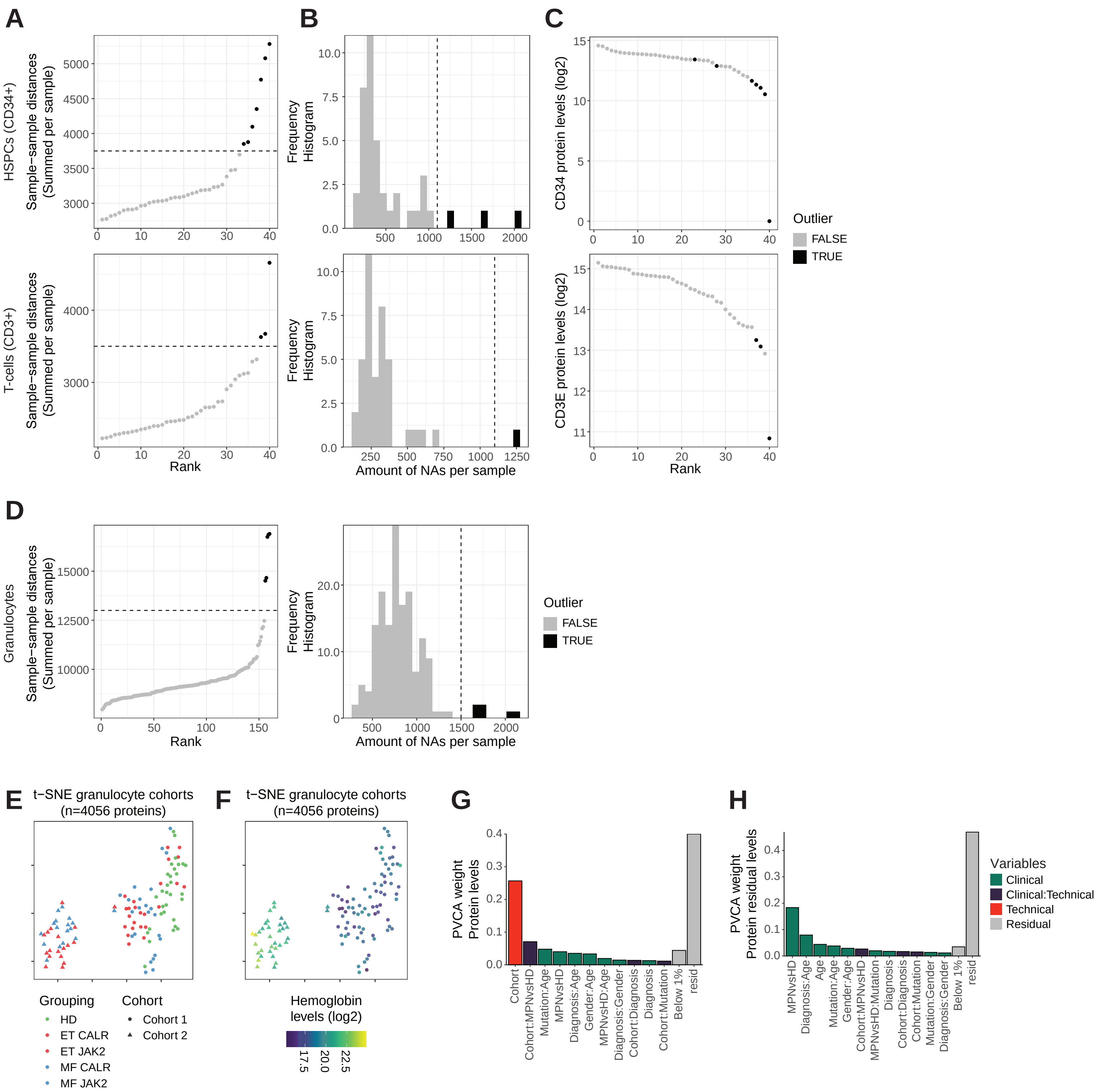

Supplementary Figure 4: Clinical proteotyping quality controls

(A) Outlier detection of the MF PBMC proteotyping of HSPCs (top) and T-cells (bottom) based on sample dissimilarities. Summed Euclidean distances of each sample compared to all other samples are shown. The dashed lines indicate the cutoffs above which samples were defined as outliers.

(B) Outlier detection of the MF PBMC proteotyping of HSPCs (top) and T-cells (bottom) based on low protein detection. Histograms show the number of not-detected proteins (NAs) per patient. The dashed lines indicate the cutoffs above which samples were defined as outliers.

(C) CD34 and CD3E protein levels across HSPC and T-cell samples, respectively, with outliers as defined in **(A)** and **(B)** marked in black.

(D) Outlier detection as in **(A)** and **(B)** for the granulocyte cohort.

(E) t-SNE embedding of granulocyte cohort proteotypes. Dimensionality reduction is based on non-regressed protein quantities (n = 4056). MPN patients and HD are labeled by disease, mutation, and cohort status.

(F) Overlay of t-SNE embedding of **(E)** with averaged hemoglobin protein levels.

(G) Principal Variance Component Analysis (PVCA) quantifying influence of selected clinical and technical variables on proteotype variance. Variables are colored according to clinical or technical nature.

(H) PVCA analysis as in **(G)** of residual protein levels after proteotype-wide regression of average hemoglobin protein levels.

Supplementary Figure 5

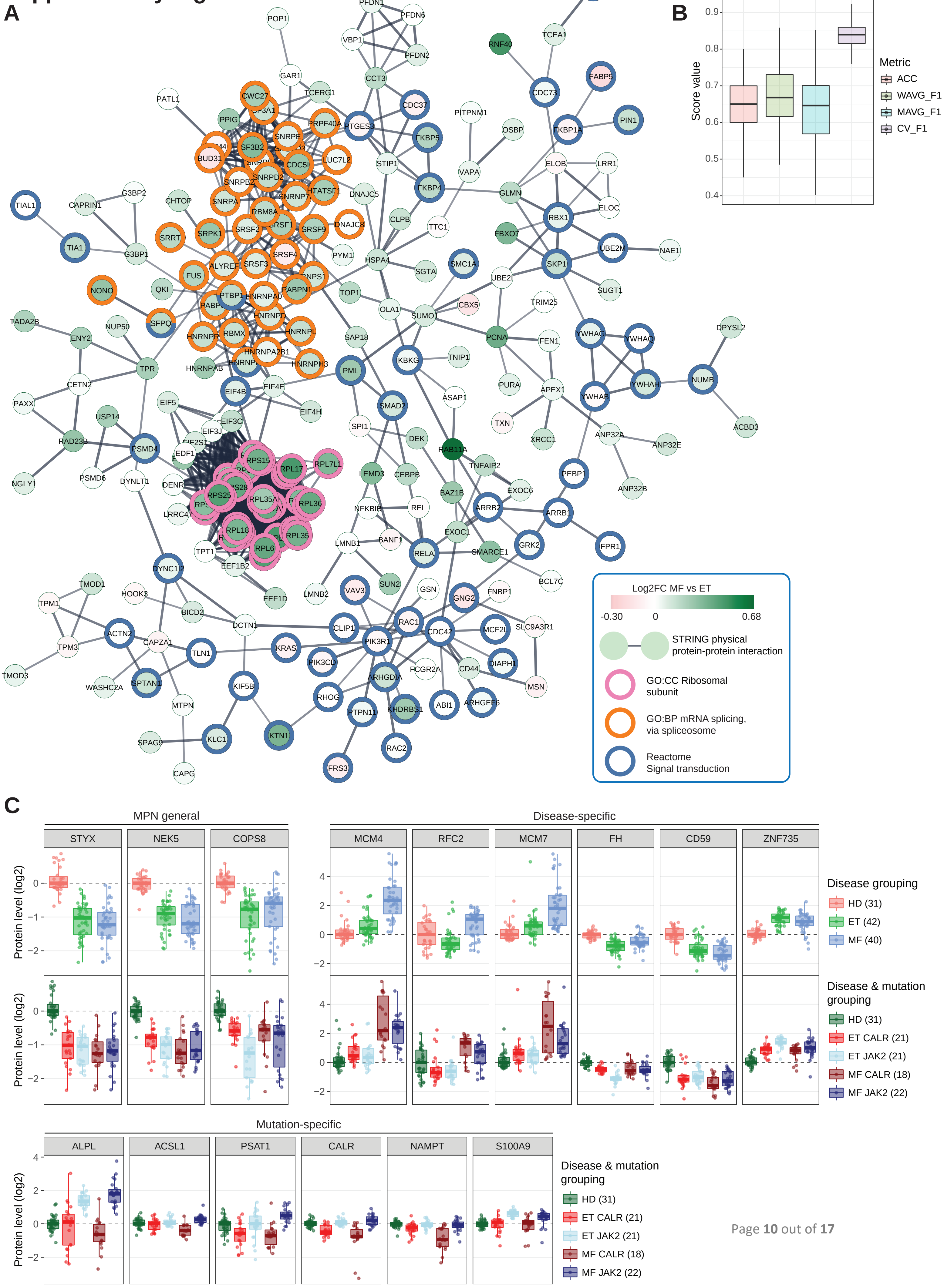

Supplementary Figure 5: Large-scale MPN granulocyte proteotyping

(A) Protein-protein interaction network of proteins significantly upregulated in ET and MF. Proteins are included that reach a significant upregulation in both ET vs HD and MF vs HD (cutoff for significance: adjusted Student's t-test p-value < 0.01). Protein node fill colors represent the fold change of MF compared to ET. Outer protein node rings indicate selected protein pathway memberships. Edges indicate protein-protein interactions (physical interaction STRING score > 0.7).

(B) Performance metric distributions of the proteotype classifier runs (n = 100). Cross-validation macro F1 (CV) is the score of the model during the training, the other three scores (test accuracy (ACC); macro F1 (MAVG); and weighted F1 (WAVG)) are calculated using the independent test dataset not used during training.

(C) Boxplots of expression levels of the 15-protein signature grouped by MPN general, disease-, and mutation-specific expression patterns. Protein levels are normalized by subtracting the median HD expression

Boxplots as in Figure 1A.

Supplementary Figure 6

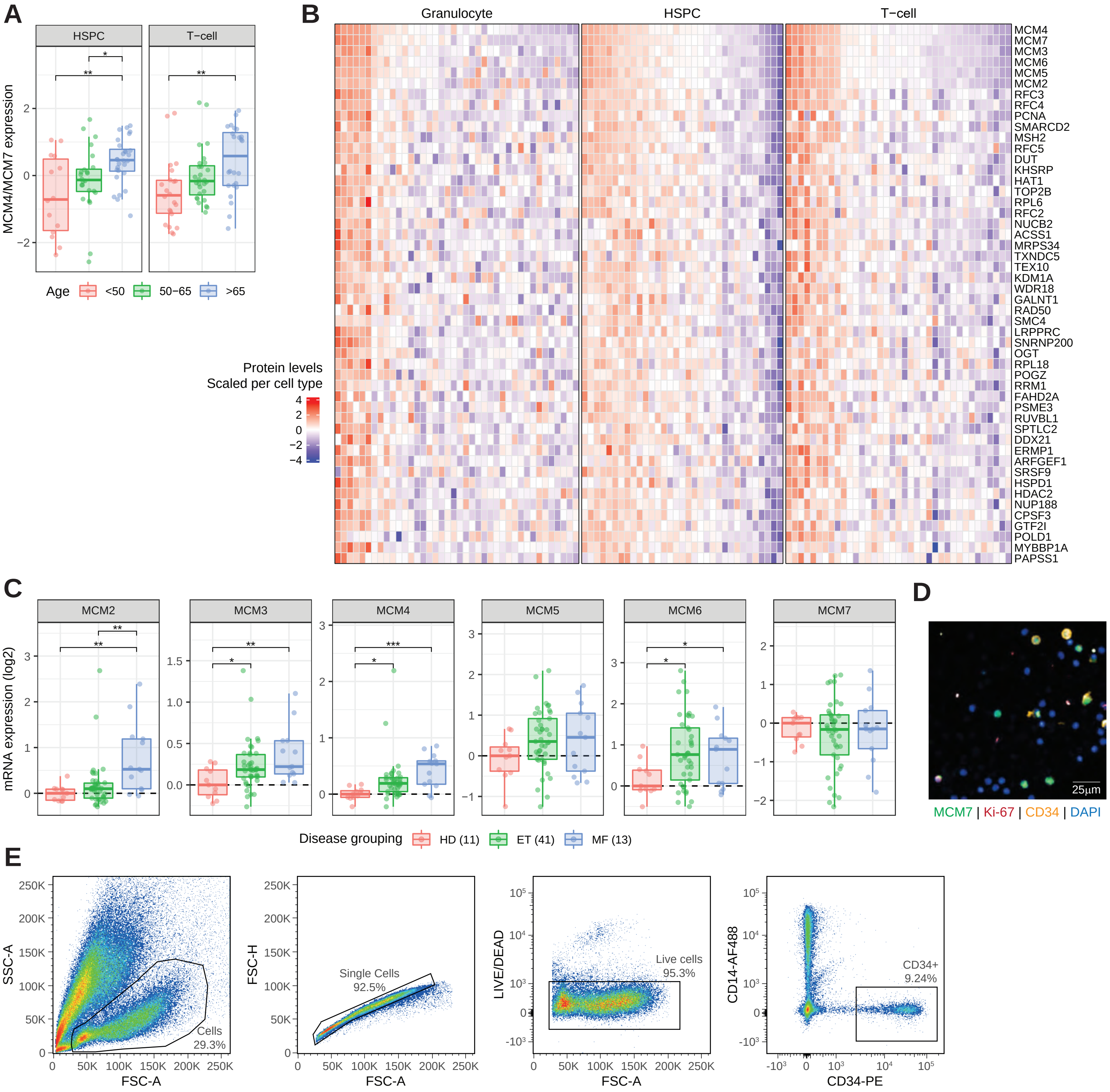

Supplementary Figure 6: Characterization of the proliferative MCM-high MF phenotype

(A) Additional boxplots to Figure 7B and C, showing patient age associating significantly to MCM4 and MCM7 levels in HSPCs and T-cells. Boxplots represent MCM4 and MCM7 protein expression levels stratified by patient age, dots represent the expression for individual MF patients (<50, n=7 and n=11; 50-65, n=12 and n=14; >65, n=14 and n=12 MF patients for HSPCs and T-cells, respectively).

(B) Heatmap of expression levels of the top-50 MCM4 and MCM7-correlating proteins across granulocytes, HSPCs, and T-cells. Proteins (rows) are ranked by averaged correlation. Patient samples (columns) are ranked by average signature expression. Protein levels are z-score normalized per protein per cell type.

(C) Microarray-based mRNA expression of MCM complex members across diseases from Rampal *et al.*, 2014 [1]. Log2 mRNA expression levels were summarized per gene by max probe selection.

(D) Representative image of MCM immunofluorescence imaging-based quantification. MCM7 (green), Ki-67 (red), CD34 (orange), and DAPI (blue) channels are shown. Representative results of two independent repeats are shown.

(E) FACS-based purification of CD34+ HSPCs for vosaroxin and carfilzomib exposure readouts as analyzed in Figure 7K and 8J.

Asterisks indicate non-adjusted two-sided Student's t-test significance: **** = $p < 0.0001$, *** = $p < 0.001$, ** = $p < 0.01$, * = $p < 0.05$; exact p-values are reported in Source Data. Boxplots as in Figure 1A.

Supplementary Figure 7

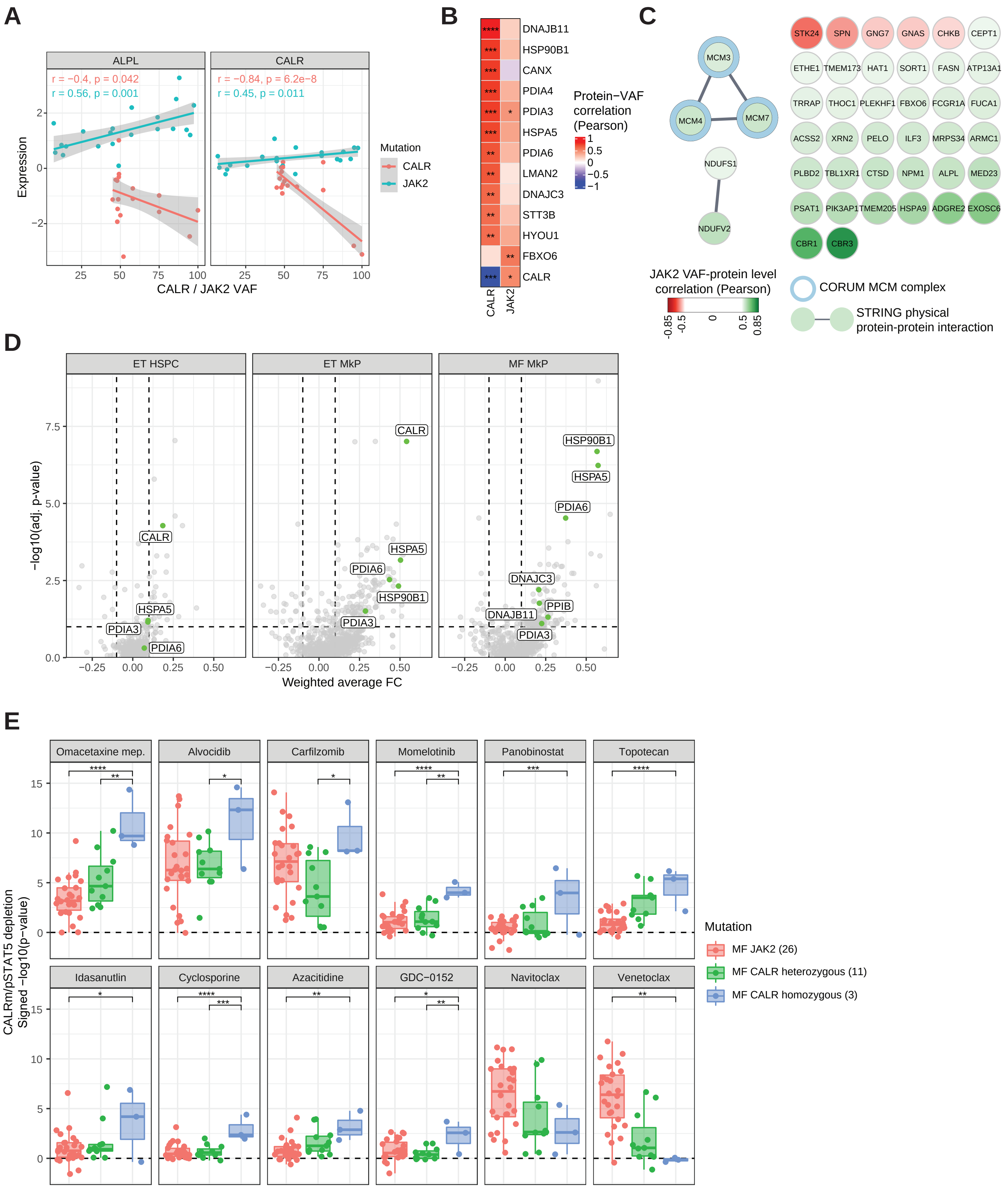

Supplementary Figure 7: Characterization of homozygous *CALR* mutation-induced ER stress

(A) ALPL and CALR protein expression levels (y-axis) compared to *CALR* and *JAK2* mutation burden (VAF; x-axis). Dots represent individual patients and dot color mutation status (MF CALR: red; MF JAK2: blue). Linear fit (solid line) and 95% confidence intervals (grey area) are shown and Pearson correlations and corresponding correlation significance are reported per mutation.

(B) Correlations between *CALR*/*JAK2* VAF and protein expression levels for detected proteins belonging to the KEGG term "Protein processing in ER". Proteins with a significant Pearson correlation ($p < 0.01$) with either *CALR* or *JAK2* VAF are shown. Colors indicate Pearson correlations, whereas asterisk indicate correlation significances.

(C) Network visualization of proteins whose expression levels correlate to *JAK2* VAF. Proteins are shown with significant Pearson correlations ($p < 0.01$). Fill colors indicate Pearson correlations, edges STRING protein-protein interactions (physical interaction STRING score > 0.7), and outer rings indicate protein memberships of the indicated complex.

(D) Validation of the ER stress signature within *CALR*-mutated ET and MF patient cells. Reanalysis of publicly available single-cell RNAseq data from Nam *et al.*, 2019 [2]. X-axis shows the weighted average FC and y-axis the adjusted p-value of *CALR*-mutated cells compared to patient-matched wild type cells from blood. MkP: megakaryocytic progenitors.

(E) Additional MF CALR homozygous-specific CALRm/pSTAT5 oncogenic drug responses as shown in Figure 8I. p-values indicate Student's t-test significance. Boxplots as in Figure 1A.

Asterisks indicate non-adjusted two-sided significance: **** = $p < 0.0001$, *** = $p < 0.001$, ** = $p < 0.01$, * = $p < 0.05$; exact p-values are reported in Source Data.

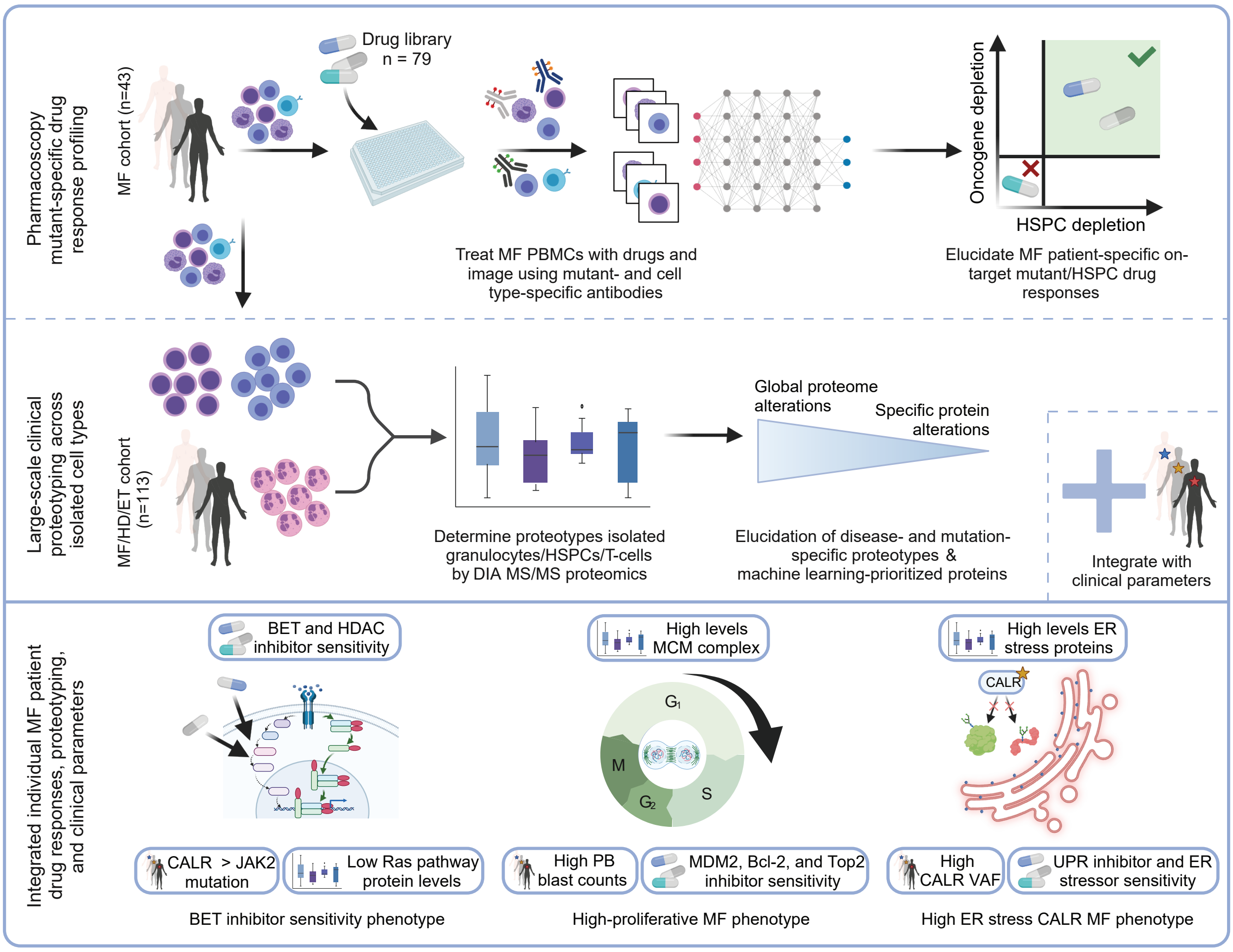

206 **Supplementary Figure 8: Graphical summary of the study workflow and key findings**

SUPPLEMENTARY TABLES

Supplementary Table S1: The full clinical annotations of the different included cohorts.

Supplementary Table S2: The processed pharmacoscopy drug response matrices.

Supplementary Table S3: The processed proteotype matrices.

SUPPLEMENTARY REFERENCES

1. Rampal, R. *et al.* Integrated genomic analysis illustrates the central role of JAK-STAT pathway activation in myeloproliferative neoplasm pathogenesis. *Blood* **123**, e123-33 (2014).

2. Nam, A. S. *et al.* Somatic mutations and cell identity linked by Genotyping of Transcriptomes. *Nature* **571**, 355–360 (2019).
