# Supplementary material for: Proteogenetic drug response profiling elucidates targetable vulnerabilities of myelofibrosis
Source: Nat Commun. 2023 Oct 12;14:6414. doi: 10.1038/s41467-023-42101-z (PMC10570306; doi:10.1038/s41467-023-42101-z)
Supplement: Supplementary file 3 — Reporting Summary [file 41467_2023_42101_MOESM3_ESM.pdf]

nature portfolio

Corresponding author(s): Berend Snijder

Last updated by author(s): Jun 10, 2023

Reporting Summary

Nature Portfolio wishes to improve the reproducibility of the work that we publish. This form provides structure for consistency and transparency in reporting. For further information on Nature Portfolio policies, see our Editorial Policies and the Editorial Policy Checklist.

Statistics

For all statistical analyses, confirm that the following items are present in the figure legend, table legend, main text, or Methods section.

n/a	Confirmed	
☐	☒	The exact sample size (*n*) for each experimental group/condition, given as a discrete number and unit of measurement
☐	☒	A statement on whether measurements were taken from distinct samples or whether the same sample was measured repeatedly
☐	☒	The statistical test(s) used AND whether they are one- or two-sided<br>*Only common tests should be described solely by name; describe more complex techniques in the Methods section.*
☐	☒	A description of all covariates tested
☐	☒	A description of any assumptions or corrections, such as tests of normality and adjustment for multiple comparisons
☐	☒	A full description of the statistical parameters including central tendency (e.g. means) or other basic estimates (e.g. regression coefficient) AND variation (e.g. standard deviation) or associated estimates of uncertainty (e.g. confidence intervals)
☐	☒	For null hypothesis testing, the test statistic (e.g. *F*, *t*, *r*) with confidence intervals, effect sizes, degrees of freedom and *P* value noted<br>*Give P values as exact values whenever suitable.*
☒	☐	For Bayesian analysis, information on the choice of priors and Markov chain Monte Carlo settings
☒	☐	For hierarchical and complex designs, identification of the appropriate level for tests and full reporting of outcomes
☐	☒	Estimates of effect sizes (e.g. Cohen's *d*, Pearson's *r*), indicating how they were calculated

Our web collection on statistics for biologists contains articles on many of the points above.

Software and code

Policy information about availability of computer code

Data collection	Raw files from LC-MS/MS proteotyping were converted to HTRMS using HTRMSConverter (Biognosys) and directly imported into Spectronaut 13.9-15.0 (Biognosys). Protein quantification and subsequent statistical analyses on DIA features extracted from Spectronaut were performed using MSStats (Choi et al., 2014) version 3.16.
Data analysis	MATLAB R2021a, RStudio 2022.02.2, R version 4.2.0, CellProfiler 2.2.0, ImageJ 1.53q, Spectronaut 15.0, MSStats version 3.16.

For manuscripts utilizing custom algorithms or software that are central to the research but not yet described in published literature, software must be made available to editors and reviewers. We strongly encourage code deposition in a community repository (e.g. GitHub). See the Nature Portfolio guidelines for submitting code & software for further information.

Data

Policy information about availability of data

All manuscripts must include a data availability statement. This statement should provide the following information, where applicable:

- Accession codes, unique identifiers, or web links for publicly available datasets
- A description of any restrictions on data availability
- For clinical datasets or third party data, please ensure that the statement adheres to our policy

The mass spectrometry proteomics data have been deposited to the ProteomeXchange Consortium via the PRIDE (Perez-Riverol et al., 2022) partner repository with the dataset identifier PXD036075. The reviewer account credentials are: username reviewer_pxd036075@ebi.ac.uk; password B7h1LrvC.

Human research participants

Policy information about studies involving human research participants and Sex and Gender in Research.

Reporting on sex and gender	The cohort represents a random sampling of the disease population. The sex of each included research participant is reported in Supplementary Table 1. Gender information was not collected as it was not relevant to the current study.
Population characteristics	Population characteristics including sex, age, and genetic alterations are reported as a metadata table in Supplementary Table 1 and visualized in Figure 2B and 5B. The first cohort consists of 17 female and 26 male donors with an age range of 22-88 (median: 62). The second cohort consists of 44 female and 68 male participants with an age range 23-95 (median: 60).
Recruitment	ET and MF patients were included who visited the University Hospital Zürich in the period of December 2012-May 2021 or the University Hospital Basel in the period March 2015-May 2019. Apart from disease diagnosis and confirmation of presence of CALR or JAK2 mutations, no patient selection criteria were imposed.
Ethics oversight	Peripheral blood samples were collected from MPN patients visiting the University Hospital Zürich in the period of April 2018-June 2019 upon written informed consent according to the Declaration of Helsinki. The study was approved by the local ethics committee (KEK-ZH-NR: 2009-0062/1 and BASEC-NR: 2018-00539). For the second cohort, the collection of blood samples and clinical data from MPN patients was approved by the Ethik Kommission Beider Basel. Written informed consent was obtained from all patients in accordance with the Declaration of Helsinki. The diagnosis of MPN was established according to the revised criteria of the World Health Organization (Arber et al., 2016). Healthy donor samples were collected from coded blood donors by the Blutspende Zürich under a study protocol approved by the Cantonal Ethics Committee, Zürich (KEK Zürich, BASEC-Nr 2019-01579).

Note that full information on the approval of the study protocol must also be provided in the manuscript.

Field-specific reporting

Please select the one below that is the best fit for your research. If you are not sure, read the appropriate sections before making your selection.

☒ Life sciences ☐ Behavioural & social sciences ☐ Ecological, evolutionary & environmental sciences

For a reference copy of the document with all sections, see nature.com/documents/nr-reporting-summary-flat.pdf

Life sciences study design

All studies must disclose on these points even when the disclosure is negative.

Sample size	113 blood samples from unique myelofibrosis patient (MF, n=43), essential thrombocythemia patients (ET, n=39), and matched health donors (HD, n=31) were subjected to proteotyping. The healthy donor cohort was age- and gender-matched to the patient cohort, and the size was chosen to allow for averaging out the biological variation between healthy individuals. An additional 43 MF patient PBMC samples were used for drug response profiling and HSPC/T-cell proteotyping. No statistical method was used to predetermine sample size. Sample sizes were selected based on previous experience to allow sufficient stratification according to disease and mutation subtypes.
Data exclusions	For high-content image-based drug screening, drug response results for 3 of the 43 patients were excluded from further analysis due to hematopoietic stem and progenitor cell (HSPC) fractions below 0.3%.
Replication	For protein quantification and subsequent statistical analyses on DIA features, in case of availability of technical replicates, analyses were run twice: on the single MS run level for QC analyses and otherwise summarized per patient or cell line for further analyses and plotting. For high-content image-based drug screening, all drugs were randomized across the plate layout with duplicates of 1uM and 10uM final concentrations, except for antibodies that had three replicates and 0.1uM, 1uM, and 10uM final concentrations. For other biological measurements and assays, a minimum of three technical or biological replicates were measured. For imaging experiments, at least two independent experiments were performed.
Randomization	For high-content image-based drug screening, to control for plate effects due to laser illumination, drugs were re-suspended as 5mM stock solutions and dispensed into the 384 well plates using an Echo 550 liquid handler (Labcyte) at their respective concentrations in a randomized plate layout.For proteotype analyses, the sample preparation and measurements were randomized for disease and mutation status. For the HSPC and T-cell proteotype analyses, additional randomization was included for common confounders such as age and sex. Otherwise, no randomization was performed as part of this study.
Blinding	The observational exploratory design of this study was not compatible with blinding.

Reporting for specific materials, systems and methods

We require information from authors about some types of materials, experimental systems and methods used in many studies. Here, indicate whether each material, system or method listed is relevant to your study. If you are not sure if a list item applies to your research, read the appropriate section before selecting a response.

Materials & experimental systems

n/a	Involved in the study
☐	☒ Antibodies
☐	☒ Eukaryotic cell lines
☒	☐ Palaeontology and archaeology
☒	☐ Animals and other organisms
☒	☐ Clinical data
☒	☐ Dual use research of concern

Methods

n/a	Involved in the study
☒	☐ ChIP-seq
☐	☒ Flow cytometry
☒	☐ MRI-based neuroimaging

Antibodies

Antibodies used	Alexa Fluor® 647 anti-CALRm (Statens Serum Institut, clone 385-06, in-house conjugated), Alexa Fluor® 647 anti-pSTAT5 (Invitrogen, #71-6900, polyclonal, in-house conjugated), Alexa Fluor® 488 anti-pSTAT5 (Invitrogen, #71-6900, polyclonal, in-house conjugated), Alexa Fluor® 488 anti-CD3 (Biolegend, #300415, clone UCHT1), Alexa Fluor® 488 anti-CD14 (Biolegend, #325610, clone HCD14), PE anti-CD34 (Biolegend, #343506, clone 581), PE anti-CALR (Abcam, #ab83220, clone FMC75), Alexa Fluor® 488 anti-GRP78 (Invitrogen, #53-9768-80, clone C38), Alexa Fluor® 488 anti-Calnexin (Abcam, #ab225061, clone EPR3632), Alexa Fluor® 594 anti-GM-130 (Abcam, #ab277236, clone EP892Y), Alexa Fluor® 647 anti-Ki-67 (Cell Signaling Technology, #12075, clone D3B5), Alexa Fluor® 647 anti-Cleaved Caspase-3 (Cell Signaling Technology, #9602, clone D3E9), anti-BrdU (BD, #347580, clone B44), Alexa® Fluor 488 anti-mouse IgG1 (Invitrogen, #A-21121, polyclonal), anti-BrdU (Abcam, #ab6326, clone BU1/75 (ICR1)), and Cy™3 Anti-Rat IgG (Jackson ImmunoResearch, #712-165-153, polyclonal).
Validation	All commercial antibodies used in this study were validated by the vendors. Please see the relevant vendors' websites for further information. The monoclonal antibody SSI-HYB 385-06 Anti Frameshift Mutated Calreticulin (Anti Crtfs), IgG1 / Kappa, clone SSI-4F10 (Art. No. 100808) (described as CALRm antibody) was developed and manufactured by Statens Serum Institut, Copenhagen, Denmark. The specificity and sensitivity of this CALRm antibody was validated in CMK11-5 cells CRISPRed to express mutant CALR (Shide et al., 2017) as well as the CALR mutant patient-derived MARIMO cell line (Kollmann et al., 2015). The CALRm antibody did not stain CALR knock-out cell lines, further validating the specificity.

Eukaryotic cell lines

Policy information about cell lines and Sex and Gender in Research

Cell line source(s)	CMK11-5, from which the wild type and the CRISPRed clone 751 were kindly provided by K. Shide (Shide et al., 2017), and K562, from which the wild type, CRISPRed mutant and knockout clone were kindly provided by A. Vannucchi. To complement the CMK background with a knockout counterpart, CMK11-5 wild-type cells were CRISPRed using a ribonucleoprotein (RNP)-based strategy (Gundry et al., 2016). After confirmation of CRISPRing in bulk isolated DNA, a single cell sort was performed. Various clones could be confirmed to be homozygous knockouts on both a DNA and protein level. CALR mutant patient-derived MARIMO cell line was as described in Kollmann et al., 2015.
Authentication	The CALR mutation status of the CMK11-5 and K562 cell lines (wild type, mutated, or knockout) was genetically verfiied. No other authantication was performed.
Mycoplasma contamination	Included cell lines were not tested for mycoplasma contamination.
Commonly misidentified lines (See ICLAC register)	No commonly misidentified cell lines as defined by the ICLAC register were used in this study.

Flow Cytometry

Plots

Confirm that:

- ☒ The axis labels state the marker and fluorochrome used (e.g. CD4-FITC).
- ☒ The axis scales are clearly visible. Include numbers along axes only for bottom left plot of group (a 'group' is an analysis of identical markers).
- ☒ All plots are contour plots with outliers or pseudocolor plots.
- ☒ A numerical value for number of cells or percentage (with statistics) is provided.

Methodology

Sample preparation	For intracellular staining of cell lines, cells were fixed and permeabilized using the Intracellular Fixation & Permeabilization Buffer Set (eBioscience) according to the manufacturer's instructions before being stained with respective antibodies. For sorting of patient PBMCs, cells were directly incubated with antibody and sorted as viable cells.

Instrument	We have used a BD FACSAria™ Fusion for sorting of patient material and a BD LSRFortessa™ for flow cytometry analysis of cell lines.
Software	FlowJo software (FlowJo Enterprise, version 10.0.8, BD Biosciences) was used for all analyses.
Cell population abundance	The imaging-based readouts that followed up on the sort confirmed purity of sorted CD34+ HSPCs.
Gating strategy	For both cell lines and patient PBMC samples, a single clear population was gated out based on FSC-A/SSC-A gating from debris. Single cells were determined based on FSC-A/FSC-H gating. Using a respectiver live/dead marker, dead cells were gated out. Finally, the resepective antibody stains were performed. Gates were defined by inclusion of controls samples with respective negative or isotype control conditions.

☒ Tick this box to confirm that a figure exemplifying the gating strategy is provided in the Supplementary Information.
