# Supplementary material for: Proteogenetic drug response profiling elucidates targetable vulnerabilities of myelofibrosis
Source: Nat Commun. 2023 Oct 12;14:6414. doi: 10.1038/s41467-023-42101-z (PMC10570306; doi:10.1038/s41467-023-42101-z)
Supplement: Supplementary file 4 — Description of Additional Supplementary Files [file 41467_2023_42101_MOESM4_ESM.pdf]

Description of Additional Supplementary Files

Supplementary Data 1
Description: Clinical data

Supplementary Data 2
Description: Pharmacoscopy Data

Supplementary Data 3
Description: Proteotyping Data
